# *Mycobacterium tuberculosis* Is Resistant to Isoniazid at a Slow Growth Rate by Single Nucleotide Polymorphisms in *katG* Codon Ser^315^

**DOI:** 10.1371/journal.pone.0138253

**Published:** 2015-09-18

**Authors:** Rose E. Jeeves, Alice A. N. Marriott, Steven T. Pullan, Kim A. Hatch, Jon C. Allnutt, Irene Freire-Martin, Charlotte L. Hendon-Dunn, Robert Watson, Adam A. Witney, Richard H. Tyler, Catherine Arnold, Philip D. Marsh, Timothy D. McHugh, Joanna Bacon

**Affiliations:** 1 Public Health England, Porton Down, Salisbury, United Kingdom; 2 St George's, University of London, Cranmer Terrace, London, United Kingdom; 3 Public Health England, Colindale, 61 Colindale Avenue, London, United Kingdom; 4 University College London, Centre for Clinical Microbiology, Royal Free Campus, Rowland Hill Street, London, United Kingdom; St. Petersburg Pasteur Institute, RUSSIAN FEDERATION

## Abstract

An important aim for improving TB treatment is to shorten the period of antibiotic therapy without increasing relapse rates or encouraging the development of antibiotic-resistant strains. In any *M*. *tuberculosis* population there is a proportion of bacteria that are drug-tolerant; this might be because of pre-existing populations of slow growing/non replicating bacteria that are protected from antibiotic action due to the expression of a phenotype that limits drug activity. We addressed this question by observing populations of either slow growing (constant 69.3h mean generation time) or fast growing bacilli (constant 23.1h mean generation time) in their response to the effects of isoniazid exposure, using controlled and defined growth in chemostats. Phenotypic differences were detected between the populations at the two growth rates including expression of efflux mechanisms and the involvement of antisense RNA/small RNA in the regulation of a drug-tolerant phenotype, which has not been explored previously for *M*. *tuberculosis*. Genotypic analyses showed that slow growing bacilli develop resistance to isoniazid through mutations specifically in *katG* codon Ser^315^ which are present in approximately 50–90% of all isoniazid-resistant clinical isolates. The fast growing bacilli persisted as a mixed population with *katG* mutations distributed throughout the gene. Mutations in *katG* codon Ser^315^ appear to have a fitness cost *in vitro* and particularly in fast growing cultures. Our results suggest a requirement for functional *katG*-encoded catalase-peroxide in the slow growers but not the fast-growing bacteria, which may explain why *katG* codon Ser^315^ mutations are favoured in the slow growing cultures.

## Introduction

The standard short-course chemotherapy for tuberculosis (TB) is prolonged; currently a 6 month period. The most important aim for improving TB treatment is to shorten the period of treatment without increasing relapse rates or encouraging the development of antibiotic-resistant strains [1]. Isoniazid together with rifampicin is at the core of the standard regimen. For isoniazid, some clinically relevant resistance mutations have been identified; primarily in *katG* and *inhA*. Approximately 50–90% of all clinical isoniazid-resistant isolates have a mutation at *katG* codon Ser^315^ [2], which encodes for the catalase-peroxidase responsible for activating the pro-drug isoniazid. Mutations in *katG* codon Ser^315^ have been shown to bypass INH activation while retaining a 50% functional catalase-peroxidase activity [3, 4]. In addition to *katG* and *inhA*, other mutated genes have been found in INH^R^ isolates [5, 6]. However, their contribution to isoniazid resistance in patients is still to be assessed. The development of antibiotic resistance can be as a result of bacterial adaptation to a hostile environment *in vivo* (other than antibiotic exposure), leading to the selection of populations of bacteria with a range of mutations beyond those defined by the site of action of the antibiotic [7]. This genetic adaptation can include compensatory mutations in metabolic processes that increase strain fitness during survival upon antibiotic exposure [8, 9]

In addition, there is a proportion of the *M*. *tuberculosis* population that is refractory to the bactericidal action of anti-tuberculosis antibiotics due to phenotypic tolerance, in which organisms are less susceptible to the action of antibiotics. This tolerance can be impacted by environmental stimuli and the subsequent physiological state of the organism [10]. It may be the result of pre-existing populations of slow growing/non replicating bacteria that are protected from antibiotic action, but it is not limited to this; it may also be due to the expression of a phenotype that limits drug activity. For example, during isoniazid exposure, epigenetic events leading to KatG-pulsing result in a pattern of dynamic persistence [11].

It remains unclear how the slow growth of *M*. *tuberculosis* contributes to isoniazid tolerance. A number of clinical studies have quantified bacteria in the sputum during the early bactericidal response of chemotherapy (EBA) and have shown bi-exponential killing [12, 13]. It is therefore possible that slow growing, metabolically inactive bacilli predominate in sputum after initial isoniazid-mediated sterilisation of the exponentially growing bacilli.

This hypothesis has been tested previously [14] and the results suggested that the cessation of the bactericidal activity of isoniazid was as a result of a rapid emergence of antibiotic resistance and not the depletion of the exponential phase growth. However, it requires further investigation as clinical data on the levels of antibiotic resistance in patients during EBA studies showed that antibiotic resistance was never encountered in isoniazid monotherapy trials [15]. We have approached the problem from a different perspective by interrogating populations of either slow growing (69.3h mean generation time (MGT)) or fast growing (23.1h MGT) bacilli in their responses to the effects of isoniazid treatment. We grew *M*. *tuberculosis* under defined and controlled conditions in steady-state in continuous culture [16, 17] and tested whether bacilli replicating at the different growth rates responded similarly to a static minimum inhibitory concentration (MIC) of isoniazid. Genotypic analyses were performed to determine the effect of different growth rates on the mutant frequency and the development of *katG* mutations. Phenotypic adaptation to isoniazid under different growth rates was also explored using RNA tiling arrays.

## Materials and Methods

### Strains and their growth

*M*. *tuberculosis* (strain H37Rv) was used in all experiments. Bacilli were enumerated on 7H10 agar plus OADC supplement.

### Continuous culture of *M*. *tuberculosis*

*M*. *tuberculosis* (strain H37Rv) was grown in chemostats under controlled conditions as described previously [16]. We cultured *M*. *tuberculosis* using CMM MOD2 [18], which contained glycerol as the limiting nutrient. Three independent continuous cultures were performed at two different growth rates to steady-state under defined and controlled conditions at pH6.9, at a temperature of 37°C and at a dissolved oxygen tension of 10% [16] [17]. The cultures achieved an MGT of 23.1h (fast growth; Cultures INH23.1, INH23.2, & INH23.3) or an MGT of 69.3h (slow growth; Cultures INH69.1, INH69.2, & INH69.3). The antibiotic was then added during steady-state at a minimum inhibitory concentration (MIC) of 0.5 mg L^-1^ to three replicate cultures at each growth rate and maintained at this level in culture throughout each time-course. Viable count, mutant frequency analyses, pyrosequencing, and transcriptomics, were performed throughout the culture time-courses, and for a minimum of 7 MGT, which is equivalent to 485.1h and 161.1h for slow growth and fast growth, respectively. At least two further fast growth or slow growth cultures were established without antibiotic exposure to provide baseline information about the differences in viability and mutation rate between the two growth rates.

### Viability measurements

The viability of the cultures was measured at each MGT using the Miles and Misra viable count method [19] with the following modification: the plate was divided into quadrants for the dilutions. In each quadrant, three 20 μl aliquots of the appropriate dilution were spotted and then left to dry at room temperature. Colonies were counted after 3 weeks incubation at 37°C.

### Mutation rate analyses

During the time-course, 6 mL culture samples were serially diluted from neat to 10^−2^. Diluted samples were plated onto Middlebrook 7H10 agar plates containing 1mg L^-1^ (2 x MIC) isoniazid in triplicate. Neat and dilute samples (10^−1^ to 10^−6^) were also plated in triplicate onto Middlebrook 7H10 agar that did not contain antibiotic, to obtain a total viable count. Colonies were counted after 3 weeks of incubation at 37°C. The mutant frequencies were calculated at each MGT by dividing the number of colonies isolated from the antibiotic plate (cfu mL^-1^) by the total viable count (cfu mL^-1^). The mutation rates were calculated for chemostats that had not been exposed to antibiotic using the mutation rate equation below.

$$\mu =\left[\left(\frac{r_{2}}{N_{2}}\right)-\left(\frac{r_{1}}{N_{1}}\right)\right]\times Ln\left(\frac{N_{2}}{N_{1}}\right)=(f_{2}-f_{1})\times Ln\left(\frac{N_{2}}{N_{1}}\right)$$

Key of terms:

μ = mutation rate

r_1_ = observed number of mutants at first time-point

r_2_ = observed number of mutants at last time-point

N_1_ and N_2_ = total numbers of viable cells at first and last time-points respectively

f_1_ and f_2_ = mutant frequencies at first and last time-points respectively

### Pyrosequencing of *katG* codon Ser^315^

Resistant colonies were isolated from slow growth (69.3h MGT; Culture INH69.3) or fast growth culture (23.1h MGT; Culture INH23.3) on plates containing 2 x MIC of isoniazid (as described above). One hundred resistant colonies were picked at random from the agar using a sterile toothpick after 6–7 MGT and after 12–13 MGT during fast growth (Culture INH23.3) and slow growth (Culture INH69.3). These colonies were emulsified in Tris-EDTA buffer and heat killed at 80°C in a heating block for 1h. Heat-killed colonies were used as a template for amplification of a 200 bp region including the *katG* codon Ser^315^ (primers: forward, CGGTCACACTTTCGGTAAGA & reverse, CCGTACAGGATCTCGAGGA) [20]. Sequence information was obtained from at least 60 colonies for each culture at each time-point. 20 μl of amplified DNA was mixed with 3 μl of streptavidin-coated Sepharose beads, 40 μl binding buffer (Qiagen 979006) and 17 μl of nuclease-free water in a 96-well microtitre plate and incubated for 5–10 minutes at room temperature while shaking at 1400 rpm. A PSQ® plate (Qiagen 979002) was prepared by adding 45 μl of 0.3 μM sequencing primer (GGACGCGATCACCA) in annealing buffer (Qiagen 979009) per well. To remove non-annealed primers and non-biotinylated product, the solution, containing the beads in suspension, was aspirated using the Vacuum Prep Tool (Qiagen 9001740). The pins were washed in 70% ethanol for 5 seconds, followed by 0.2 M NaOH for 5 seconds to denature the DNA and finally washing buffer (Qiagen 979008) for 5 seconds. The vacuum was then released and the tool was placed in the PSQ®96 plate containing 45 μl 0.3 μM sequencing primer in annealing buffer and gently shaken to release the beads with the single-stranded PCR product attached. The sequencing primer was annealed to the PCR product by heating the plate at 80°C for 2 minutes and then allowing it to cool down to room temperature. The dispensation order was designed to cover the region of the single nucleotide polymorphism (SNP) directly after the sequencing primer and the region after this to confirm the presence of wild type (WT) sequences. To sequence the SNP, nucleotides A, C, T and G were added at this position. The enzyme mix, substrate mix, and the four deoxynucleotides were added to the PSQ®96 cartridge (Qiagen 979004). The PSQ®96 plate and cartridge were loaded into the PSQ®96 MA instrument for sequencing.

### Sanger sequencing of *katG*

The heat killed colonies used for pyrosequencing were also used as a source of template DNA for PCR amplification of Rv1908c (*katG*) using primers katG1 (CACGCGGGGTCTGACAAA) and katG2 (GACGAGGCGGAGGTAATCTA). A 2249 bp region of the H37Rv genome (2153774 to 2156223) was amplified, which included *katG* and approximately 100bp upstream and downstream. High fidelity polymerase KAPA HiFi HotStart ReadyMix (Kapa Biosystems KK2601) was used as per the manufacturer’s instructions. The PCR comprised of 3 mins at 95°C followed by 35 cycles at 98°C for 20 sec, 68°C for 15 sec and 72°C for 1 min 15 sec. The presence of an appropriate amplified DNA fragment was confirmed by agarose gel electrophoresis and the amplicons were purified using QIAquick PCR Purification Kit (Qiagen 28104). The resulting amplicons were submitted to Eurofins Genomics (Ebersberg, Germany) for Sanger sequencing using primers katG1 and katG2 as well as a further internal primer katG3 (TATTGGGGCAAGGAAGCCAC). Sequences (Phred score >20) were assembled using SeqMan Pro (DNASTAR Lasergene 11). The resulting consensus obtained for each isolate was compared to gene *katG* (Rv1908c) from the H37Rv genome (Genbank accession: AL123456.3), to generate a SNP report. The DNA sequences were translated and the resulting protein sequences aligned to the KatG protein (GenBank accession no: CCP44675.1) using MegAlign Pro (DNASTAR Lasergene 11) in order to identify the resulting amino acid changes.

### RNA extraction and purification

20 mL of bacterial culture was sampled from two chemostat cultures for each growth rate; an MGT of 23.1h (fast growth; Cultures INH23.2, & INH23.3) and an MGT of 69.3h (slow growth; Cultures INH69.2, & INH69.3), prior to antibiotic addition and after 2 MGT (at approximately 138.6h and 46.2h for the slow growers and fast growers, respectively). Samples were added to 4 volumes of guanidine thiocyanate (GTC) lysis solution (5M GTC; 5% lauryl sarcosine; 25mM Tri-sodium citrate; 0.5% Tween 80) and incubated at room temperature for 1h. The sample mixture was centrifuged for 15 minutes at 1935 x g and the supernatant discarded. The pellets were re-suspended in 1.2 ml of Trizol (Invitrogen 15596018) and mixed thoroughly. The sample was transferred to a 2 ml tube containing 0.5 ml of 0.1 mm silica beads (Fisher Scientific MBR-247-105B) and lysed using a reciprocal shaker (FastPrep FP120) for 45 seconds at a speed of 6.5. The supernatant was transferred into a tube containing 240 μl of chloroform and shaken vigorously for 20 seconds. This solution was centrifuged at 2415 x g for 10 minutes. The aqueous phase was removed and added to 600 μl chloroform, shaken vigorously for 20 seconds and centrifuged for 10 minutes at 2415 x g twice. Following the second chloroform precipitation, the aqueous phase was added to 600 μl isopropanol plus 60 μl sodium acetate (Sigma Aldrich S7899) and frozen at -70°C at least overnight. Total RNA was isolated from the extractions using the mirVana™ miRNA Isolation kit (Agilent AM1561) and DNase I-treated using the DNA-*free*™ kit (Ambion® AM1906) as per the manufacturer’s instructions. RNA was quantified using a nanodrop 3000 and the quality assessed on an Agilent 2100 bioanalyzer (Agilent Technologies, CA, USA with an Agilent RNA 6000 Nano Kit (Agilent, 5067–1511).

### RNA labelling

RNA was labelled using the Kreatech ULS™ Fluorescent Labeling Kit for Agilent arrays (Kreatech EA-023). The labelled RNA was fragmented by adding 2 μl 10x fragmentation buffer, incubating for 15 minutes at 70°C, then adding 2 μl stop solution (Ambion® AM8740). Labelled RNA (20 μl) was added to 27.5 μl Kreatech blocking reagent (Kreatech EA-023), 55 μl of 2x Hybridisation buffer and 7.5 μl of molecular grade water. Arrays were hybridised overnight at 65°C, then washed in Gene Expression wash buffer 1 (Agilent 5188–5327) for 1 minute at room temperature with agitation, then in Gene Expression wash buffer 2 for 1 minute at 37°C with agitation. Slides were scanned immediately using an Agilent Scanner.

### Transcriptomic analyses

Whole genome gene expression analyses were performed. Microarray experiments were performed using a custom Agilent tiling array (ArrayExpress accession A-BUGS-47) with 180,000 60-mer oligos evenly tiled across the *M*. *tuberculosis* H37Rv genome. Features were extracted from the array images using Agilent Feature Extraction Software (v10.7) with local background correction. Probes were first filtered to only include those covering annotated genes. Intensity values were normalised and analysed using GeneSpring software (version 12.6 GX). Firstly, quantile normalisation was applied across the combined slow and fast growth rate datasets, followed by an averaging of the expression level of all probes across each open reading frame, on either the sense (S) or antisense (AS) strand. A 2-way ANOVA (using a Benjamini-Hochberg correction p-value of P = 0.05 and growth rate and MGT as conditions) was used to identify significantly differentially expressed genes between fast growth rates and slow growth rates either in the presence or absence of isoniazid. A further filter was applied to select genes with at least a two-fold change in gene expression. Gene lists derived from all pairwise comparisons can be found in the supporting information (S1 Table Gene lists for all pair-wise comparisons). Genes were assigned groups based on their entries in Tuberculist (http://tuberculist.epfl.ch/).

For the analysis of the expression of small and non-coding RNAs, Limma [21] analyses were performed following quantile normalisation between arrays to find differentially expressed regions (oligos) within the genome. Ratios of fluorescence intensity values across the genome were visualised using a custom version of the genome browser JBrowse (http://www.ncbi.nlm.nih.gov/pubmed/19570905) which included the novel tracks MultiBigWig and MultiXYPlot (Source code and data available at https://github.com/rtylerr/jbrowse1.11-MultiBigWig). The differentially expressed regions identified by Limma could then be visualised alongside the calculated ratio plots for manual curation.

### Real–time quantitative PCR (RTq-PCR)

PCR reactions utilised the 16S rRNA gene as an endogenous control. The *ahpC* primer and probe sequences were designed using Primer3 software (http://primer3.ut.ee/). Primers and probes were optimised to have a reaction efficiency of 90–110% to validate them for use with the ΔΔCt method of quantification; these data can be found in the supporting information along with primer and probe sequences (S2 Table RT-PCR reaction efficiencies and primers). Reverse transcription took place in a total volume of 20 μl containing 100 ng total RNA, 300 ng random primers (Invitrogen^TM^ Life Technologies), 10 mM DTT, 0.5 mM each of dCTP, dATP, dGTP and dTTP, and 200 units Superscript III (Invitrogen^TM^ Life Technologies). The 20 μl reactions were assembled in a standard ABI 96-well plate in triplicate as follows; 1.8 μl of 10 μM *ahpC* primers or 1 μl of 10 μM 16S primers, 0.5 μl of 10 μM of probe, 10 μl of Taqman Universal Mastermix II with UNG (Applied Biosystems part No 4428175) and 1 μl of cDNA. 16S rRNA was used as an endogenous control. Reactions were performed in an ABI-7900 lightcycler with the programme as follows; 50°C for 2 minutes, 95°C for 10 minutes, 40 cycles of 95°C for 15 seconds and 55°C for 45 seconds. Data were analysed using the SDS 2.4 and RQ Manager 1.2.1 Software (ABI) to calculate ΔΔCt and relative quantification values.

## Results and Discussion

### The response to isoniazid exposure in *M*. *tuberculosis* varies with growth rate

**Fig 1:**
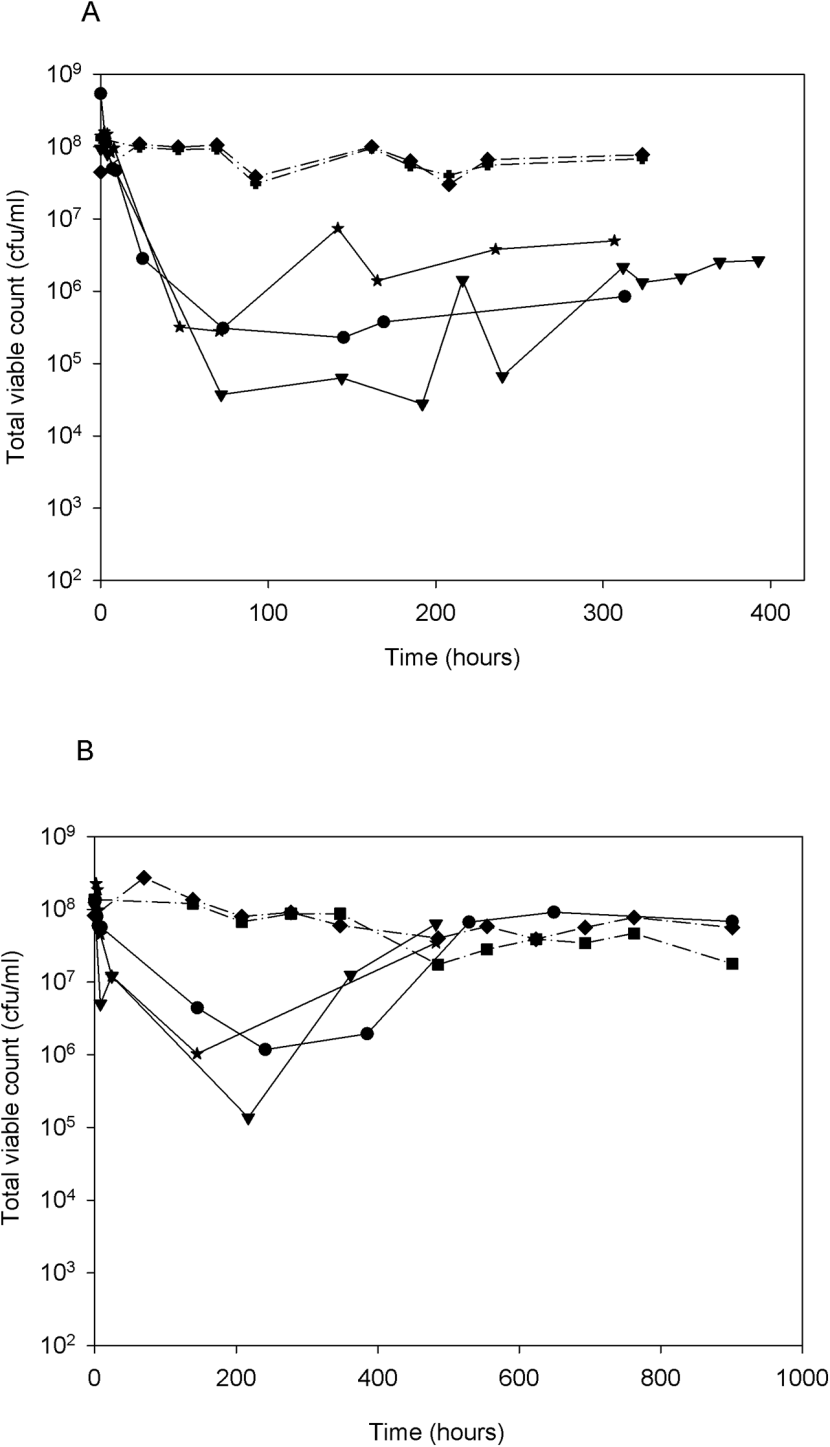
The viability of *Mycobacterium tuberculosis* H37Rv growing at either a fast growth rate or a slow growth rate in response to isoniazid exposure. The viability of *Mycobacterium tuberculosis* H37Rv growing at either a fast growth rate (Panel A; stars: Culture INH23.1, circles: Culture INH23.2, & triangles: Culture INH23.3) or a slow growth rate (Panel B; stars: Culture INH69.1, triangles: Culture INH69.2, & circles: Culture INH69.3) for at least 7 MGT in response to isoniazid (0.5 mg L^-1^) added at 0h and continuously throughout culture. Each line represents viable count data from an individual chemostat culture. The dotted lines in each case are control cultures without isoniazid addition.

We assessed whether bacilli replicating at two different growth rates of 23.1h MGT or 69.3h MGT responded similarly to static and inhibitory levels of isoniazid. This was to address the hypothesis that the sub-population of organisms that persist through treatment is predominantly slow growing. For the first 2–3 MGT after antibiotic exposure, there was no difference in the bactericidal effect of isoniazid at slow and fast growth rates, both giving approximately a 10^3^ cfu mL^-1^ drop in viable organisms by 2–3 MGT (Fig 1). The slow growing cultures recovered and increased their growth rate temporarily to a faster rate than imposed previously to re-establish a cell titre of 10^8^ cfu ml^-1^ whereas fast growing cultures (23.1h MGT) retained a stable level of viable bacilli at 10^5^–10^6^ cfu mL^-1^ (Fig 1). This bactericidal response, followed by a plateau in the cell number, has been observed previously with isoniazid exposure in a number of studies, particularly in metabolically inactive or stationary phase cultures [14, 22, 23, 24]. However, it has not been possible until now using continuous culture to show a clear difference between the responses of slow and fast growing organisms to isoniazid. This response reflects some aspects of the EBA studies, which demonstrated that during therapy, the decrease in the bacterial population in sputum was a biphasic response [13] with the rapid bacillary decrease observed during the first 2 days of therapy thought to be associated with killing of fast growing bacteria. The second phase in treatment, which is mediated by rifampicin and pyrazinamide, and causes a much slower decrease in bacterial density between days 3 and 14 is thought to target organisms that are growing more slowly.

We observed a contrasting response between bacilli dividing at the two growth rates following an initial drop in viability. Growth rate in continuous culture is controlled by the flow rate of a limiting nutrient, in this case glycerol [18]. Such a substantial drop in the number of viable bacteria at both growth rates will have resulted in a transient and brief excess of glycerol in the growth medium. This provided the nutrient resource for an increase in growth rate for both the slow and the fast growers. However, only the slow growing cultures (69.3h MGT) were able to increase their growth rate and re-establish the original cell number present prior to antibiotic addition. The most likely explanation is that the fast growers were dividing at a rate very close to the maximum physiologically achievable growth rate (in this system; μ_max_), as it has been determined previously that increasing the growth rate of fast growth cultures, to a rate that is faster than 23.1h MGT resulted in wash-out of the cultures (data not shown). If this were the case then an increase in the growth rate (and therefore an increase in biomass) would not be possible, even in the presence of excess glycerol.

### Mutant frequencies increased at both fast and slow growth rates in the presence of isoniazid

**Fig 2:**
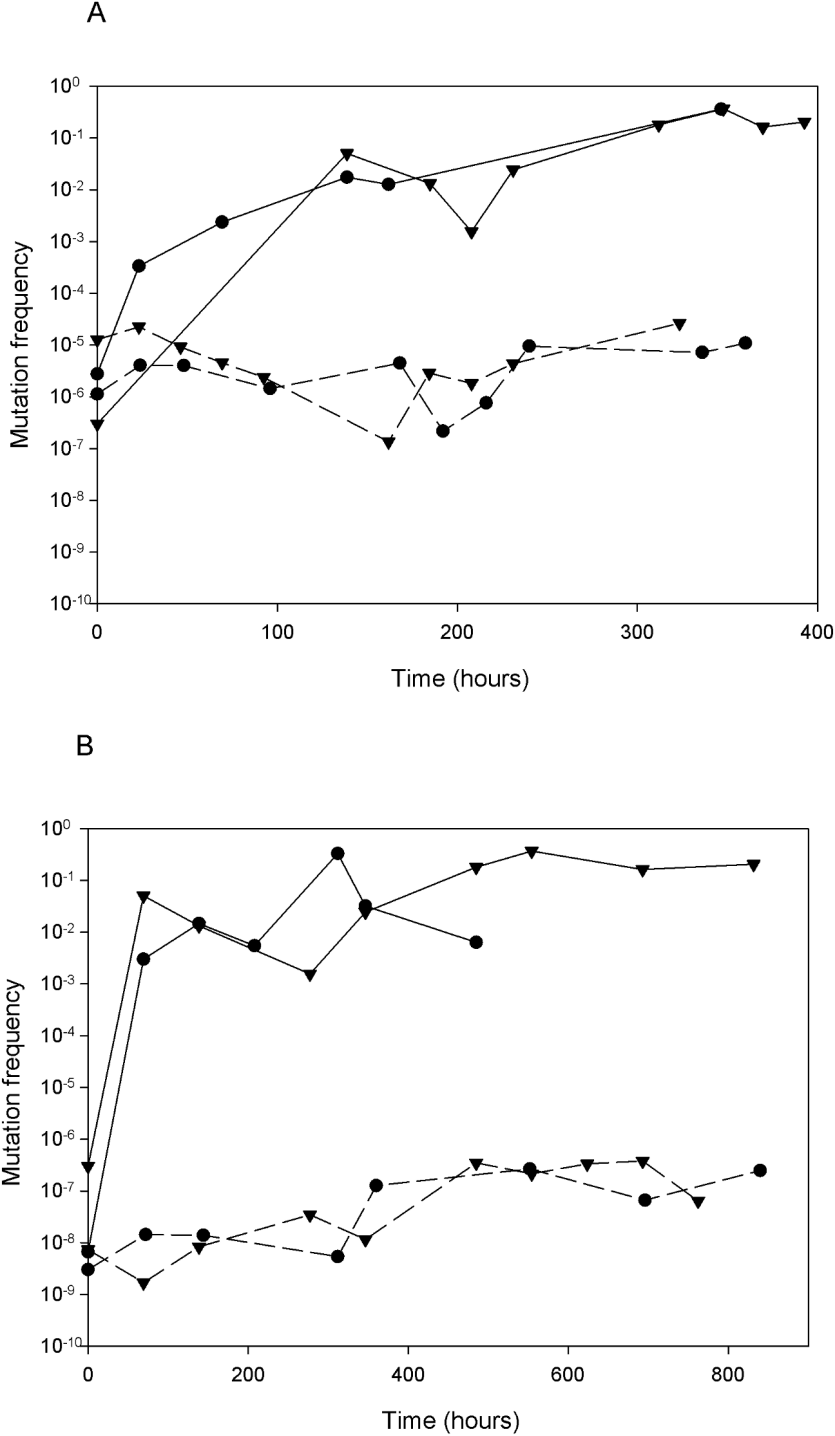
The mutant frequency of *Mycobacterium tuberculosis* H37Rv growing at either a fast growth rate or a slow growth rate in response to isoniazid exposure. The mutant frequency of *Mycobacterium tuberculosis* H37Rv growing at either a fast growth rate (Panel A, solid lines; circles: Culture INH23.2 & triangles: Culture INH23.3) or a slow growth rate (Panel B solid lines; circles: Culture INH69.2 & triangles: Culture INH69.3) for at least 7 MGT in response to isoniazid (0.5 mg L^-1^) added at 0h and continuously throughout culture. Each line represents the mutant frequency from an individual chemostat culture. The dotted lines in each case are control cultures without isoniazid addition

It was hypothesised that the different growth recovery profiles of the fast and slow growing cultures following isoniazid exposure could be due to a difference in the mutant frequencies between the two growth rates, and so these frequencies were measured. In the antibiotic-free control cultures, the mutant frequencies remained consistent throughout the time-course, but were consistently higher under fast growth conditions (Fig 2). It was observed previously that the proportion of mutants in continuous logarithmic cultures in complex medium increased with a shortening of the MGT/increase in growth rate [25, 26]. This could be due to an increased mutation rate caused by the stress imposed upon cells forced to divide more frequently. In our study, the mutation rates were calculated in the absence of antibiotic at between 0 MGT and 6–7 MGT using the accumulation method described previously [27]. Mutation rates in replicate fast and slow growing cultures were similar (5.78 x 10^−6^ and 7.00 x 10^−7^ for slow growth conditions (69.3h MGT) and 6.12 x 10^−7^ and 3.80 x 10^−6^ for fast growth conditions (23.1h MGT). Although mutation rates have been reported as being lower during latent infection in patients [28] during which growth rates are slower by definition, Ford *et al*., 2011 [29] observed that in non-human primates there was very little difference in the mutation rates between active disease, latent disease, and reactivated disease. Our results show that mutation rates were not significantly affected by growth rate under the conditions tested once a steady-state has been established. However, there was a higher mutant frequency observed in our fast growth rate cultures than in the slow growth rate cultures during steady-state. Therefore, during the initial growth phase prior to steady-state there was likely to be a differential increase in the mutation rate between the two growth rates. The mutant frequency increased, in both fast and slow growing cultures in the presence of isoniazid, due to the selective pressure imposed both in culture and on selective agar. In 2007, Gumbo *et al*., [14] used a hollow fibre pharmacodynamic model to simulate time-related changes in the bacterial population (using doses of isoniazid that reflected levels given to patients). The characteristic plateau in cell number was observed and coincided with increased mutant frequency with regard to *katG* mutations. This indicated that the bactericidal activity of isoniazid was being arrested by the emergence of resistance mutations and not a reduction in the log-phase populations. Our work shows that an increase in the mutant frequency coincided with the recovery from cell death. Cells growing exponentially at either fast or slow growth rate were initially killed and culture viability dropped as cells were continually removed from the system; fast-growth-rate cultures then recovered to at least the imposed growth rate and maintained consistent culture viability whilst slow growers recovered their cell number via an increased growth rate to a level observed prior to antibiotic addition. In both cases the recovery was coincident with a substantial increase in mutant frequency (Fig 2). This supports the observations of Gumbo *et al*. [14] that the accumulation of resistant mutant populations (in both slow and fast growers) was in part responsible for the development of persisting populations of bacilli.

### Slow growth is associated with increased heterogeneity in *katG* codon Ser^315^

Various mutations in the *katG* gene have been reported in isoniazid-resistant isolates, the most common mutations are within *katG* codon Ser^315^, which is reported as present in 50–90% of all isoniazid-resistant isolates and is associated with high-level resistance [2, 30]. Three common SNPs were identified in *katG* codon Ser^315^ and all of these mutations could be found in the slow growing populations exposed to isoniazid (Table 1). The percentage of *katG* codon Ser^315^ mutants selected under slow growth in the presence of isoniazid was at 95% after 12 MGT. Between 7 MGT and 12 MGT the AAC codon within the *katG* codon Ser^315^ locus remained consistently at about 30% of the *katG* mutant population and gave an amino acid change to asparagine. The *katG* codon Ser^315^ mutation that increased in number over time in the slow growth cultures was ACC, which rose from 19% to 37%. In fast growing populations, the majority (≥ 86%) of mutant colonies isolated were WT at the *katG* codon Ser^315^ locus after 6 MGT and after 13 MGT (Table 1).

**Table 1:** Singe nucleotide polymorphisms within the *katG* codon Ser^315^ in populations of isoniazid-resistant *Mycobacterium tuberculosis* H37Rv.

*katG* codon Ser^315^ sequence (amino acid change)		Culture condition and time-point of sample (MGT from time zero)				
	INH69.3 7MGTpyro	INH69.312 MGTpyro	INH69.3 12 MGTsanger	INH23.36 MGTpyro	INH23.313MGTpyro	INH23.313MGTsanger
AGC (WT)	9	4	0	59	86	77 (**65**)
AAC (S315N)	33	33	27	0	2	2
AGA (S315R)	3	6	5	0	6	7
ATC (S315I)	19	20	29	1	1	1
AGG (S315R)	0	0	0	0	1	1
ACC (S315T)	19	28	37	0	1	1
Mixed	14	8	0	0	1	0
Putative duplication/partial deletion						11
Total number	97	99	100	60	98	100

The number of singe nucleotide polymorphisms within the *katG* codon Ser^315^ in populations of isoniazid-resistant *Mycobacterium tuberculosis* H37Rv colonies that were isolated from continuous cultures growing either at a slow growth rate (Culture INH69.3) or fast growth rate (Culture INH23.3) that had been exposed to isoniazid. Colonies were selected on agar containing 2 x MIC isoniazid (1 mg L^-1^). One hundred colonies were picked after 6-7MGT and 12–13 MGT and were pyrosequenced at the *katG* codon Ser^315^. The total number of colonies, at each time-point, for which sequence information was obtained, is shown here. Colonies picked from Culture INH69.3 and Culture INH23.3 at 12 MGT or 13 MGT, respectively, were also subjected to Sanger sequencing and 65 (shown in bold; further detail in B) out of the 77 colonies that were found to be WT for the *katG* codon Ser^315^ were mutated elsewhere in the *katG* gene. Further detail on these mutations can be found in Table 2. Eleven colonies were found to have a putative duplication and partial deletion within *katG* in the fast growth culture, INH23.3, and the sequence data could not be fully interpreted.

Time-point 12–13 MGT for slow growth cultures and fast growth cultures was interrogated further using Sanger sequencing to verify the observation that 86% of fast growing cells were WT at *katG* codon Ser^315^ using pyrosequencing (Table 1). A range of alternative *katG* mutations was observed in 65 out of 77 of the fast growing colonies that were WT at Ser^315^ (Table 2), some of which have been described previously [3, 5, 30–33]. The resistance genotype in the remaining 12 WT colonies was not confirmed. Five colonies at each growth rate were found to contain a double mutation in *katG*; some of these double mutations included a mutation at *katG* codon Ser^315^ (Table 3) and most of the alternative mutations caused a frameshift upstream of *katG* codon Ser^315^. Similarly to the study by Gumbo *et al*., [14], our studies have shown that a *katG* mutant population emerges at the point at which bactericidal activity arrests and persistence commences (at around 2 MGT). We have been able to add further information to these previous findings [14] by showing that *katG* codon Ser^315^ mutants could have been preferentially selected for during slow growth. Contrary to the idea that the slow growing bacilli are the predominant sub-population that persists through treatment via antibiotic tolerance mechanisms and not via well characterised resistance mutations, we found that slow growing cells develop antibiotic resistance mutations in *katG* codon Ser^315^. Although fast growing populations maintained replication in the presence of isoniazid and the mutant frequencies of the two growth rates were equivalent, there were very few mutations in *katG* codon Ser^315^ in the fast growing cells. A previous study has also shown a lack of clinically relevant mutations *in vitro* in logarithmic growth phase cultures [34]. These data suggest that there are factors other than mutant frequency that can drive the evolution of isoniazid resistance in *M*. *tuberculosis*.

**Table 2:** Mutations identified in *katG* under fast or slow growth rates including mutations in *katG* codon Ser^315^.

Mutation	AA change
G1C	M1L
T2C	M1A
G3A	M1V
G3C	M1V
G3T	M1V
3Gdel	Frameshift
7GAdel	Frameshift
18Ains	Frameshift
19Cins	Frameshift
G28T	E10*
54Cins	Frameshift
78GAAATACins	Frameshift
G91T	E31*
G113A	W38*
140Tins	Frameshift
G241T	E81*
G264T	Q88H
295Gdel	Frameshift
G311A	R104Q
G320A	W107*
355Cins	Frameshift
G356C	R119P
G362A	G121D
367Gins	Frameshift
371Gdel	Frameshift
A413G	N138S
A425C	D142A
G451T	V151F
G452A	W198*
[597Gins]	Frameshift
628Ains	Frameshift
633Gins	Frameshift
T685G	Y229D
[G944A]	S315N
[G944C]	S315T
[G944T]	S315I
C945G	S315R
[C945A]	S315R
A970C	T324P
G983T	W328L
1102Tins	Frameshift
C1153T	R385W
G1282C	G428R
G1282T	G428W
[G1283T]	G428V
{1317Gdel}	Frameshift
A1316G	Q439R
1559Cdel	Frameshift
1701Tins	Frameshift
G1795T	G599*
T1985G	L662R
T2002C	W668R
G2095A	G699R

Colonies picked from slow growth (Culture INH69.3) and fast growth culture (Culture INH23.3) after 12–13 MGT were subjected to Sanger sequencing and found to contain *katG* mutations other than those found in *katG* codon Ser^315^. These mutants included 65 out of the 77 colonies that were previously found to be WT for the *katG* codon Ser^315^ but were mutated elsewhere in the *katG* gene. The notation used for each mutation is as follows: original nucleotide followed by the nucleotide number and finally the new nucleotide. The corresponding amino acid changes or effects at each position are also indicated. In cases where an amino acid alteration is indicated by an asterisk there was a truncation of the gene. The square brackets indicate that the mutation was found in both growth rates, curly brackets indicate a mutation found only during slow growth and no brackets indicate a mutation only found during fast growth.

**Table 3:** Double nucleotide polymorphisms within the *katG* gene.

Culture name; time-point	Alternative mutation; nucleotide position & (codon Ser^315^)	No. of colonies with this mutation	Amino acid change or effect
INH69.3; 12MGT	1317Gdel (ATC)	1	Frameshift (S315I)
	G1283T (ATC)	1	G428V (S315I)
	597Gins (AAC)	3	Frameshift (S315N)
INH23.3; 13MGT	A1316G & G1795T	1	Q439R & G599*
	1559Cdel (AGA)	1	Frameshift (S315R)
	597Gins (AAC)	1	Frameshift (S315N)
	597Gins (AAC)	1	Frameshift (S315N)
	355Cins & G264T	1	Frameshift & Q88H

Double nucleotide polymorphisms within the *katG* gene in colonies isolated from slow growth (Culture INH69.3) and fast growth cultures (Culture INH23.3) after 12–13 MGT were identified by Sanger sequencing. Five colonies at each growth rate were found to contain a double mutation in *katG*; some of these double mutations included a mutation at *katG* codon Ser^315^, which are indicated in brackets. The notation used for each mutation is as follows: original nucleotide followed by the nucleotide number and finally the new nucleotide. Some of the *katG* codon Ser^315^ mutations will have shifted by a nucleotide because of a deletion (“del”) or insertion (“ins”) upstream of the *katG* codon Ser^315^. Corresponding amino acid changes or effects at each position are also indicated. In cases where an amino acid alteration is indicated by an asterisk there was a truncation of the gene.

Genetic mutations that confer antibiotic resistance can have differing effects upon bacterial fitness, resulting in a reduced growth rate [35]. One interpretation of our data would be that *katG* codon Ser^315^ mutations have a fitness cost, that reduced the organism’s ability to grow at an imposed MGT of 23h but not at 69h; the latter population may have been dividing slowly enough (even during a growth spurt at 2MGT) at the latter growth rate to accommodate the fitness cost of *katG* codon Ser^315^ mutations, so that they became the predominate mutations. Another possible explanation for the predominance of *katG* codon Ser^315^ mutations in the slow growing population is that the slow growth rate was selecting against the survival of mutants with the alternative sites of mutation observed. SNPs within *katG* codon Ser^315^ still allow for catalase-peroxidase activity and resistance to isoniazid is likely to occur through a slowing in the rate of the superoxide-dependent activation of isoniazid. In contrast to this, many of the mutations (frameshifts and deletions) found in the fast growing population have disrupted the function of *katG* entirely [36].This points to a requirement for functional *katG*-encoded catalase-peroxide in the slow growers but not the fast-growing bacteria. Further characterisation of the mutant population prior to drug-addition would inform as to whether the mutation diversity was already reduced in the slow growth cultures prior to selection with isoniazid and aid interpretation of the role of KatG in growth rate-specific responses to isoniazid.

A high proportion of *katG* codon Ser^315^ mutations in our slow growers were replaced by Thr^315^ (ACC, S315T, Table 1). This is the most commonly occurring *katG* mutation, where S315T, is associated with clinically significant levels of isoniazid resistance. Previously, the S315T mutant was shown to retain its virulence in the mouse model of the disease. The interpretation at the time was that a significant loss of bacterial fitness did not result from this common mutation [37]. Combined with our findings, these data indicate that one explanation is that a fitness cost is associated with S315T, which only accommodates slow growing mycobacteria. As S315T is predominant in both our slow growing cultures and in the patient population, we suggest that phenotypes of transmitted isoniazid-resistant strains are most likely to be slow growing populations of *M*. *tuberculosis*.

### The effect of growth rate on global RNA expression prior to isoniazid exposure

The whole genome transcriptional responses (including non-coding/small RNA (sRNA)) to isoniazid exposure were analysed over the first 2 MGT to identify differential expression under the two growth rates. The expression profiles for slow growth and fast growth cultures in the absence of antibiotic were very similar, with only 11 genes showing differential expression (Table 4 & Table 5). Four genes were expressed at a significantly higher level in fast growth compared to slow growth (Rv1592c, Rv0196, Rv2429, and Rv2428) and seven genes were expressed at a significantly higher level under slow growth (Rv0820-AS, Rv2638, Rv2781c-AS, Rv2392-AS, Rv3572, Rv3089, Rv1139c-AS), some of which were up-regulated on the antisense RNA strand (AS) to the gene. Differentially expressed genes (post-antibiotic exposure), were assigned a functional group that was obtained from the Tuberculist database (http://tuberculist.epfl.ch/). The percentages of gene functions in each group are shown for down-regulated genes and up-regulated genes under each growth rate (S3 Table Genes that were significantly differentially regulated between 0 and 2 MGT in fast and slow cultures).

**Table 4:** Genes that were more highly expressed during fast growth compared with slow growth prior to isoniazid addition.

Rv no.	Genename	Predicted function	P-value
Rv1592c-S		Conserved hypothetical protein	0.007014
Rv0196-S		Transcriptional regulatory protein	0.006672
Rv2429-S	*ahpD*	Alkyl hydroperoxide reductase	0.006672
Rv2428-S	*ahpC*	Alkyl hydroperoxide reductase subunit C	0.006528

Genes that were more highly expressed by at least two-fold during fast growth compared with slow growth prior to isoniazid addition. AS and S indicate whether the higher levels of transcript were detected in the sense or antisense strand respectively.

**Table 5:** Genes that were more highly expressed during slow growth compared with fast growth prior to isoniazid addition.

Rv no.	Genename	Predicted function	P-value
Rv0820-AS	*phoT*	Phosphate-transport ATP-binding protein	0.006528
Rv2638-S		Conserved hypothetical protein	0.001306
Rv2781c-AS		Unknown function	0.016715
Rv2392-AS	*cysH*	3'-phosphoadenosine 5'-phosphosulfate reductase	0.007014
Rv3572-S		Conserved hypothetical protein	0.007448
Rv3089-S	*fadD13*	Fatty Acid-CoA-Ligase	0.007014
Rv1139c-AS		Conserved hypothetical membrane protein	0.006948

Genes that were more highly expressed by at least two-fold in slow growth compared with fast growth prior to isoniazid addition. AS and S indicate whether the higher levels of transcript were detected in the sense or antisense strand respectively.

## The Effect of Growth Rate on Global RNA Expression Following Isoniazid Exposure

### Genes differentially expressed under both growth rates

There were 140 genes that were up-regulated at both growth rates between 0 and 2 MGT post-isoniazid additions (S3 Table Genes that were significantly differentially regulated between 0 and 2 MGT in fast and slow cultures). Genes that encode NADH dehydrogenases (nuo operon, NADH dehydrogenase I) were significantly down-regulated at both growth rates, potentially decreasing the production of NAD^+^ and reducing the pool of NAD+ for isoniazid binding. Changes in NADH metabolism may confer some antibiotic tolerance to isoniazid; indeed increasing the NADH/NAD^+^ ratio has been shown to protect InhA against inhibition by the isoniazid-NAD adduct formed upon isoniazid activation [38]. We conclude that a down-regulation of NADH dehydrogenases could increase the NADH cellular concentration, which competitively inhibits the binding of isoniazid-NAD adduct to InhA.

**Fig 3:**
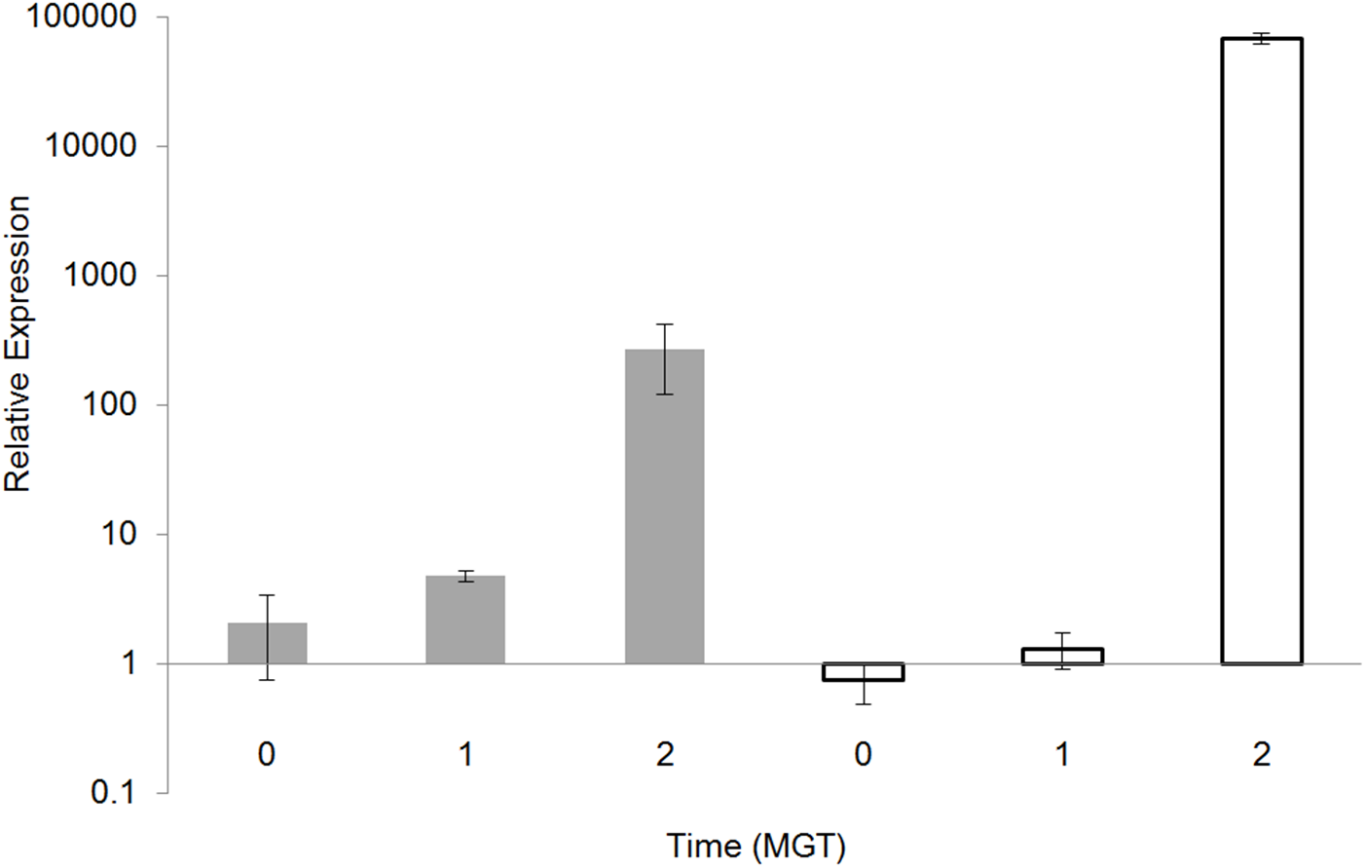
The expression of *ahpC* transcript. The relative expression of *ahpC* transcript for *Mycobacterium tuberculosis* H37Rv growing at either a fast growth rate (grey bars) or a slow growth rate (open bars) at 0h 1MGT, & 2MGT post-isoniazid addition measured using qRT-PCR. Relative expression was calculated using the ΔΔCt method, following MIQE guidelines, with normalisation to the endogenous control 16sRNA.

The expression of *ahpC* (alkyl hydroperoxide reductase subunit C) RNA was more than two fold higher in fast growing compared to slow growing cultures prior to antibiotic exposure. Following isoniazid addition the expression of both *ahpC* and *ahpD* (alkyl hydroperoxide reductase subunit D) increased over time at both growth rates. Both of these genes were shown to be induced by isoniazid exposure in a previous study [39]. The expression level of *ahpC* was higher in slow growth by 2 MGT compared to fast growth, and this was further confirmed using qRT-PCR (Fig 3). Catalase/peroxidases protect *M*. *tuberculosis* during oxidative stress by detoxifying hydrogen peroxide, reactive nitrogen intermediates and reducing organic peroxides. The peroxidase activity encoded for by *katG* is essential for the activation of isoniazid. As mentioned above, mutations in the *katG* codon Ser^315^ result in a moderate reduction of isoniazid activation, but the enzyme can still protect against host antibacterial radicals; albeit with reduced activity [3, 4]. Previously, in both isoniazid-resistant *katG*-deficient strains and isoniazid-susceptible strains that were exposed to isoniazid, there was enhanced expression of AhpC (alkyl hydroperoxide reductase), so in the fast growers, increased levels of AhpC could be compensating for a loss of catalase-peroxidase activity in mutants possessing alternative *katG* mutations [30, 40]. The requirement for increased *ahpC* expression during slow growth, after isoniazid exposure, could be explained by a need to protect against the additive effects of free radical isoniazid intermediates [41] and increased exposure to free radicals through a longer replicative life span [42], which could also explain the dependency of slow growers on functional KatG catalase-peroxidase. This display of growth rate-dependent stringency in the selection of *katG* codon Ser^315^ mutations warrants further investigation. What also remains unclear is why *ahpC* was at a higher transcriptional level in the fast growing cells prior to isoniazid addition; this may be central to understanding the differences in the post-antibiotic response that followed.

Two further genes up-regulated at both growth rates were Rv1592c (unknown function; also expressed at a higher level at fast growth pre-antibiotic exposure) and Rv3065 (*mmr*), which encodes for an efflux pump that has previously been shown to be significantly over-expressed in *M*. *tuberculosis* exposed to isoniazid [43]. As with *ahpC*, the antisense RNA strand to *mmr* was also up-regulated in the slow growth culture. Of the genes involved in mycolic acid biosynthesis [44], Rv0470c (cyclopropane mycolic acid synthase 2) was up-regulated at both growth rates.

### Genes differentially expressed under fast growth

Of the 73 genes that were up-regulated at a fast growth rate and not at a slow growth rate, 10% were involved in lipid metabolism (S4 Table The functional group assigned to genes that were significantly differentially regulated). Four of the up-regulated genes encoded for lipoproteins (Rv0179c, Rv1244, Rv1690, and Rv3016). They have a diverse range of functions and their role in adaptation to antibiotic exposure is still unclear. However, two were up-regulated in a previous study upon isoniazid exposure [45]. There are examples of lipoproteins contributing to resistance to cell wall inhibitors, such as PrsA and oxacillin resistance in *Staphylococcus aureus* [46].

Other genes up-regulated include Rv1029c (*fbpC* antigen 85C, trehalose dimycolate-cord factor), Rv2846c (*efpA*; efflux pump, antibiotic detoxification), Rv1854c (*ndh* NADH dehydrogenase), and Rv1772 (unknown function), all of which have previously been induced upon isoniazid exposure [39, 45]. Gene, Rv2763c (*dfrA*, dihydrofolate reductase (DHFR), was significantly down-regulated following isoniazid exposure during fast growth. Three mycolic acid synthases (Rv0560c, Rv3119, and Rv0990) were expressed at a lower level in fast growing bacilli compared with those growing slowly, and this could also be an example of a mechanism to reduce the availability of a target site for isoniazid. In addition to the six *mce*-family genes down-regulated at both growth rates, the expression level of a further 5 *mce*-family genes (Rv0291, Rv0299, Rv0424c, Rv0461 and Rv3208) was reduced overtime at a fast growth rate.

### Efflux pump, *efpA*

Gene *efpA* was up-regulated in fast-growing bacilli, and not in slow growing cells, during isoniazid exposure in our study. The specific condition under which this gene has been induced suggests that its expression is modulated by growth rate and serves as a means of persistence in fast growing organisms. A proportion of isoniazid-resistant *M*. *tuberculosis* clinical isolates do not have mutations in any of the genes associated with resistance to the antibiotic [24]. This suggests that other mechanism(s) may be involved, possibly including efflux proteins that are capable of pumping the drug out of the cell. It has been shown, in a number of microarray studies, that *M*. *tuberculosis* responds to isoniazid exposure by up-regulating the gene expression of these efflux pumps [39, 45, 47, 48]. There are five efflux pumps in total (Rv2459, Rv1819c, Rv3728, Rv3065, and Rv2846c) that have been characterised for isoniazid action. Gene Rv2846c (*efpA*) has been shown to be specific for isoniazid efflux, with the aid of efflux inhibitors, and presents itself as an alternative/complementary isoniazid resistance mechanism to the classical mutations that are usually observed [49]. Previously, it has been demonstrated, using the inhibitor reserpine, that some strains are dependent upon the action of efflux pumps for their isoniazid resistance [14, 24, 50]. Their studies suggested that efflux pump inhibitors can reverse mycobacterial tolerance to isoniazid. All these findings, including our data, indicate that there may be a role for efflux pump inhibitors in the treatment of TB, as they also have proven utility to treat multidrug-resistant TB. Further varied growth rate studies that either block efflux mechanisms (using CCCP) or used an *efpA* mutant would go some way to determining the importance of this gene for survival of fast growth organisms during isoniazid stress [51].

### Differential expression of antisense RNA in slow growing *M*. *tuberculosis*

At a slow growth rate, a significantly high proportion (72%) of up-regulated RNA expression was antisense to known or putative genes with functions in protein secretion, toxin-antitoxins, and mammalian cell entry [52–55]. The role of toxin-antitoxins in the adaptation of *M*. *tuberculosis* to antibiotic has not been investigated previously; but they have the potential for regulating replication under stressful conditions, which would include control of persister cell populations [56]. Mycolic acid biosynthetic genes, Rv1819c (mycolic acid ABC transporter protein) and Rv0469 (*umaA*; mycolic acid synthase) were also down-regulated. Very few genes [38] were down-regulated at a slow growth rate alone. It could be that the slow growing organisms have down-regulated many of their cellular processes by the binding of complementary cis-antisense RNA, providing a transitory, more sensitive, approach for controlling mechanisms in times of stress including regulation of genes *ahpC* and *mmr*. The sRNA B55 was identified previously as being up-regulated in response to oxidative stress [57] and here we show that it was expressed under slow growth rate conditions prior to antibiotic addition (Table 6). Both sRNAs G2 and MTS1082 are down-regulated over time at a slow growth rate during antibiotic exposure.

**Table 6:** sRNA that were differentially expressed in *M*. *tuberculosis* exposed to isoniazid.

						Slow growth (69.3h MGT)			Fast Growth (23.1h MGT)		
Name of sRNA	Alternative name	Start site	End	Orientation	Reference	0 MGT	1MGT	2MGT	0 MGT	1MGT	2MGT
B11	Mpr19, MTS2822	4099478	4099386	-	[52, 53]	+	+	+	+	+	+
B55	MTS0479	704187	704247	+	[52]	+	-	-	-	-	-
C8	Mcr6	4168281	4168154	-	[52, 53]	+	+	+	+	+	+
F6	Mcr14, MTS0194, Mpr13	293604	293641	+	[52, 53]	-	-	-	-	-	-
G2	MTS1310	1915190	1914962	-	[53]	-	-	0	-	-	+
Mcr19		575033	575069	+	[52]	0	0	0	0	0	-
Mpr4	MTS2823	4100669	4100968	+	[54]	-	-	-	-	-	-
MTS1338		1960667	1960783	+	[54]	-	-	-	-	-	-
MTS1082		1547129	1547268	+	[54]	-	-	0	-	-	-
Aspks		2299745	2299886	+	[53]	+	+	+	+	+	+
Aspks		2305814	2305955	+	[53]	+	+	+	+	+	+
AS1726		1952291	1952503	-	[53]	-	-	-	-	-	-
Mcr7		2692172	2692521	+	[52]	-	-	-	-	-	-
Mcr11	MTS0997, ncrMT1302	1413094	1413224	-	[54, 55]	+	+	+	+	+	+

*M*. *tuberculosis* grown at an MGT of either 23.1h (fast growth) or 69.3h (slow growth) was exposed to an MIC level (0.5 mg L^-1^) of isoniazid. LIMMA analysis was used to identify differentially expressed sRNA in response to isoniazid exposure at these two different growth rates and these were scored as ‘expressed’ if they were greater than 2 standard deviations from the mean intensity. sRNA expressed on the sense strand (+), sRNA expressed on the antisense strand (-), and sRNA not expressed on either strand (0).

## Concluding Remarks

Continuous culture of *M*. *tuberculosis* has enabled us to control growth rate and study the impact of slow or fast growth on the organism’s response to isoniazid. Through transcriptome analyses we have identified phenotypic differences between the two growth rates that include efflux mechanisms and the involvement of antisense RNA and sRNA; their role in the regulation of a drug-tolerant phenotype needs further exploration. Contrary to the idea that the slow growers are the predominant sub-population that exhibit tolerance to isoniazid-treatment through phenotypic mechanisms and not via resistance mutations, we found that slow growing bacilli develop resistance to isoniazid though mutations specifically in *katG* codon Ser^315^. Our findings indicate that there is a fitness cost associated with *katG* codon Ser^315^ mutations that is only accommodated by slow growing mycobacteria. We conclude that the recovery (in cell density) of the slow growing cultures but not the fast growing bacilli was due to increased growth rate and a more homogeneous isoniazid-resistant population that consists predominantly of *katG* codon Ser^315^ mutants. In contrast, the fast growing population consisted of a mixture of *katG* mutations that are less frequently encountered in clinical samples and would result in an inactive catalase-peroxidase. Slow growing *M*. *tuberculosis* seemed to have a greater requirement for a *katG*-encoded catalase-peroxidase and this may be due to the additive effect of free radical INH intermediates combined with exposure to free radicals through a longer replicative life span. As the proportion of S315T is high in both our slow growing continuous cultures and in the patient population, we suggest that the predominant phenotype of the transmitted isoniazid-resistant clinical *M*. *tuberculosis* strains reflects that of slow growing mycobacteria. This is critical information for the design and implementation of antibiotic treatments for TB, particularly in the *in vitro* preclinical evaluation of antibiotics against *M*. *tuberculosis*.

## Supporting Information

S1 Table Gene lists for all pair-wise comparisons. (XLSX)

S2 Table RT-PCR reaction efficiencies and primers. (XLSX)

S3 Table Genes that were significantly differentially regulated between 0 and 2 MGT in fast and slow cultures. (DOC)

S4 Table The functional group assigned to genes that were significantly differentially regulated. (XLSX)
