# Supplementary material for: Mycobacterium tuberculosis Is Resistant to Isoniazid at a Slow Growth Rate by Single Nucleotide Polymorphisms in katG Codon Ser315
Source: PLoS One. 2015 Sep 18;10(9):e0138253. doi: 10.1371/journal.pone.0138253 (PMC4575197; doi:10.1371/journal.pone.0138253)
Supplement: S3 Table — (DOC) [file pone.0138253.s003.doc]

| **Down-regulated over time** | | | | | | | | | |  | |
| --- | --- | --- | --- | --- | --- | --- | --- | --- | --- | --- | --- |
| **Fast Growth** | | | **% genes** | **Slow Growth** | | **% genes** | **Both Growth Rates** | **% genes** | | |  |
| Amino Acid Biosynthesis | | | 4.95 | ABC Transporter | | 2.44 | Amino Acid Biosynthesis | 5.62 | | |  |
| Central and Secondary Metabolism | | | 8.91 | Central and Secondary Metabolism | | 17.07 | cell division | 2.25 | | |  |
| DNA Modification | | | 0.99 | DNA Modification | | 4.88 | Central and Secondary Metabolism | 21.35 | | |  |
| DNA Replication | | | 1.98 | Fatty Acid and Lipid Metabolism | | 9.76 | Chaperone Proteins | 2.25 | | |  |
| Fatty Acid and Lipid Metabolism | | | 5.94 | PE/PEGRS | | 4.88 | CRISPR | 1.12 | | |  |
| Mammalian Cell Entry | | | 4.95 | Protein Synthesis | | 7.32 | Fatty Acid and Lipid Metabolism | 10.11 | | |  |
| PE/PEGRS | | | 3.96 | Toxin-antitoxin | | 2.44 | Mammalian Cell Entry | 6.74 | | |  |
| Protein Synthesis | | | 0.99 | Transcription Factors | | 7.32 | MSCRAMM Binding | 2.25 | | |  |
| Toxin-antitoxin | | | 0.99 | Unknown Function | | 36.59 | Protein Secretion | 3.37 | | |  |
| Transcription Factors | | | 0.99 | Membrane Proteins (Function unknown) | | 4.88 | Stress Response | 2.25 | | |  |
| Transposase | | | 0.99 | misc. | | 2.44 | Transcription Factors | 2.25 | | |  |
| Unknown Function | | | 23.76 |  | |  | Transposase | 2.25 | | |  |
| Membrane Proteins (Function unknown) | | | 33.66 |  | |  | Unknown Function | 24.72 | | |  |
| Insertion element IS6110 uncharacterized 12.0 kDa protein | | | 0.99 |  | |  | Membrane Proteins (Function unknown) | 7.87 | | |  |
| misc. | | | 5.94 |  | |  | misc. | 5.62 | | |  |
|  | | | **Table S4**  A) Genes that were significantly differentially regulated between 0 and 2 MGT in fast and slow cultures; assigned a function group |  | |  |  |  | | |  |
| **Up-regulated over time** | | | | | | | | |  | | |
| **Fast Growth** | **% genes** | **Slow Growth** | | | **% genes** | **Both Growth Rates** | | | **% genes** | | |
| ABC Transporter | 1.37 | ABC Transporter | | | 1.22 | ABC Transporter | | | 1.43 | | |
| Amino Acid Biosynthesis | 1.37 | Amino Acid Biosynthesis | | | 1.22 | Amino Acid Biosynthesis | | | 0.71 | | |
| Central and Secondary Metabolism | 5.48 | Central and Secondary Metabolism | | | 12.80 | Central and Secondary Metabolism | | | 7.14 | | |
| DNA Modification | 1.37 | Chaperone Proteins | | | 0.61 | DNA modification | | | 1.43 | | |
| Efflux pump | 1.37 | CRISPR | | | 1.83 | DNA replication | | | 1.43 | | |
| Fatty Acid and Lipid Metabolism | 10.96 | DNA Modification | | | 0.61 | Fatty Acid and Lipid Metabolism | | | 8.57 | | |
| PE/PEGRS | 6.85 | Fatty Acid and Lipid Metabolism | | | 6.10 | Mammalian Cell Entry | | | 1.43 | | |
| Protein Secretion | 1.37 | Mammalian Cell Entry | | | 1.83 | Multidrug Resistance (MMR) Proteins | | | 0.71 | | |
| Toxin-antitoxin | 1.37 | Multidrug Resistance (MMR) Proteins | | | 0.61 | PE/PEGRS | | | 5.71 | | |
| Transcription Factors | 6.85 | MSCRAMM Binding | | | 0.61 | Polysachharide Biosynthesis | | | 2.14 | | |
| Transposase | 5.48 | PE/PEGRS | | | 9.76 | Protein Secretion | | | 1.43 | | |
| Unknown Function | 43.84 | Protein Secretion | | | 4.27 | Protein Synthesis | | | 1.43 | | |
| Membrane Proteins (Function unknown) | 6.85 | Protein Synthesis | | | 3.05 | Stress Response | | | 1.43 | | |
| misc. | 5.48 | Stress Response | | | 1.22 | Toxin-antitoxin | | | 4.29 | | |
|  |  | Toxin-antitoxin | | | 3.66 | Transcription Factors | | | 5.71 | | |
|  |  | Transcription Factors | | | 4.88 | Transposase | | | 1.43 | | |
|  |  | Transposase | | | 3.05 | Unknown Function | | | 30.00 | | |
|  |  | Unknown Function | | | 34.15 | Insertion element IS6110 uncharacterized 12.0 kDa protein | | | 12.14 | | |
|  |  | Membrane Proteins (Function unknown) | | | 5.49 | Membrane Proteins (Function unknown) | | | 5.71 | | |
|  |  | misc. | | | 3.05 | misc. | | | 5.71 | | |

B)

**Table S4** Genes that were significantly differentially regulated between 0 and 2 MGT in fast and slow cultures post-isoniazid exposure were assigned a function group that was obtained from the Tuberculist database (http://tuberculist.epfl.ch/). The percentages of genes in each functional group are shown for down-regulated genes (Panel A) and up-regulated genes (Panel B) under each growth rate condition.
